# A dual H-type tracheoesophageal fistula; why not being repaired simultaneously? A case report and review of literature

**DOI:** 10.1186/s12887-023-03945-y

**Published:** 2023-06-19

**Authors:** Amirhossein Hosseini, Reza Sinaei, Mehrnoush Hassas Yeganeh, Masoud Ghanbari Boroujeni, Naghi Dara, Saeed Sadr, Abolfazl Iranikhah, Mohsen Rouzrokh

**Affiliations:** 1 Pediatric Gastroenterology, Hepatology and Nutrition Research Center, Research Institute for Children’s Health, Shahid Beheshti University of Medical Sciences, Tehran, Iran; 2 Department of Pediatrics, School of medicine, Kerman University of Medical Sciences, Kerman, Iran; 3 Clinical Research Development Unit, Afzalipour Hospital, Kerman University of Medical Sciences, Kerman, Iran; 4 Shahid Beheshti University of Medical Sciences, Tehran, Iran; 5 Shahid Beheshti University of Medical Sciences, Medical School, Tehran, Iran; 6 Research Institute for Children’s Health, Shahid Beheshti University of Medical Sciences, Pediatric Gastroenterology, Hepatology and Nutrition, Tehran, Iran; 7 Mofid Children’s Hospital, Department of Pediatric Pulmonology, Shahid Beheshti University of Medical Sciences, Tehran, Iran; 8 Qom University of Medical Sciences, Pediatric Gastroenterology, Hepatology and Clinical Nutrition, Qom, Iran; 9 Pediatric Surgery Research Center, Research Institute for Children’s Health, Shahid Beheshti University of Medical Sciences, Pediatric Surgery, Tehran, Iran

**Keywords:** Tracheoesophageal fistula, Infant, Surgery, H-type fistula, Double fistula, Flexible bronchoscopy

## Abstract

**Background:**

H-type Tracheoesophageal Fistula (TEF) is a particular type of congenital esophageal anomalies, in which patients present with non-specific symptoms that can result in delayed diagnosis. Here, we report two pediatric cases with a rarer variant called ‟dual H-type TEFˮ.

**Case presentation:**

We present two cases of H-type TEF. The first was a 45-day-old boy with feeding problem and cyanosis while feeding, and the second was a three-month-old girl with cough and choking after feeding from the first day of birth. In both cases, two separate TEFs were detected during diagnostic evaluation by flexible bronchoscopy. Both were repaired simultaneously through a cervical incision. The first patient deteriorated 13 days after the surgery, disturbancing in acid-base balance and expired unfortunately.

**Conclusion:**

Hence, it is necessary to consider the possibility of double TEF in any newly diagnosed H-type TEF.

## Background

Isolated Tracheoesophageal Fistula (TEF) or H-type TEF accounts for approximately 4% of congenital esophageal anomalies [1]. In this anomaly, esophageal atresia does not exist [2]. It has a better prognosis among different types of TEF and is not associated with other congenital malformations [3]. The manifestations of this anomaly are non-specific, consisting of choking during feeding, cyanotic spells, recurrent coughs, abdominal distention, recurrent chest infections and pneumonia (typically in the right upper lobe) [1, 2, 4]. Diagnosis can be established after an upper gastrointestinal contrast study and flexible bronchoscopy [2, 3]. This anomaly can be repaired through surgery with a favorable survival rate [2].

Double and H-type TEF can present major diagnostic and management difficulties. Herein, we present two cases of dual H-type TEF. In both cases, two separate TEFs were detected during diagnostic evaluation and both were repaired simultaneously through a cervical incision.

## Case 1

**Fig. 1:**
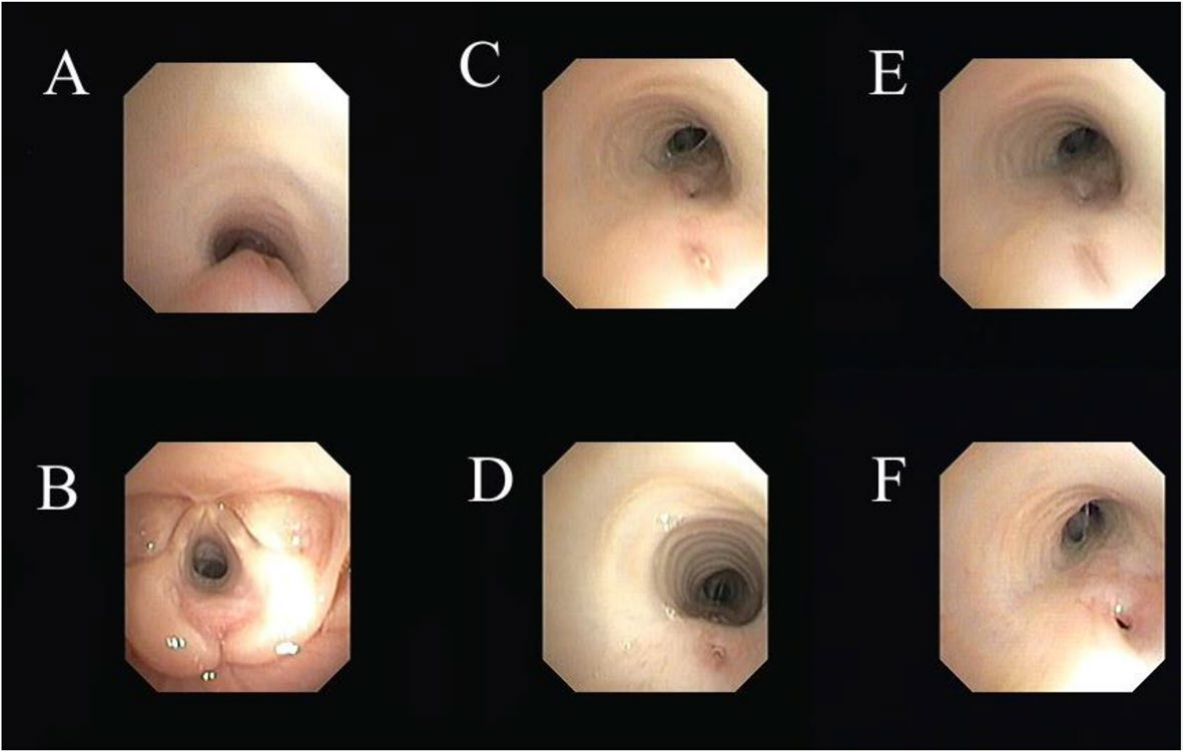
**A** Severe tracheomalacia in the proximal part of the trachea. **B** LTEC grade 2. **C-F** Double H-type tracheoesophageal fistula. Fistulas were 1 cm distant from each other, were located below the vocal cords, and were extrathoracic

**Fig. 2:**
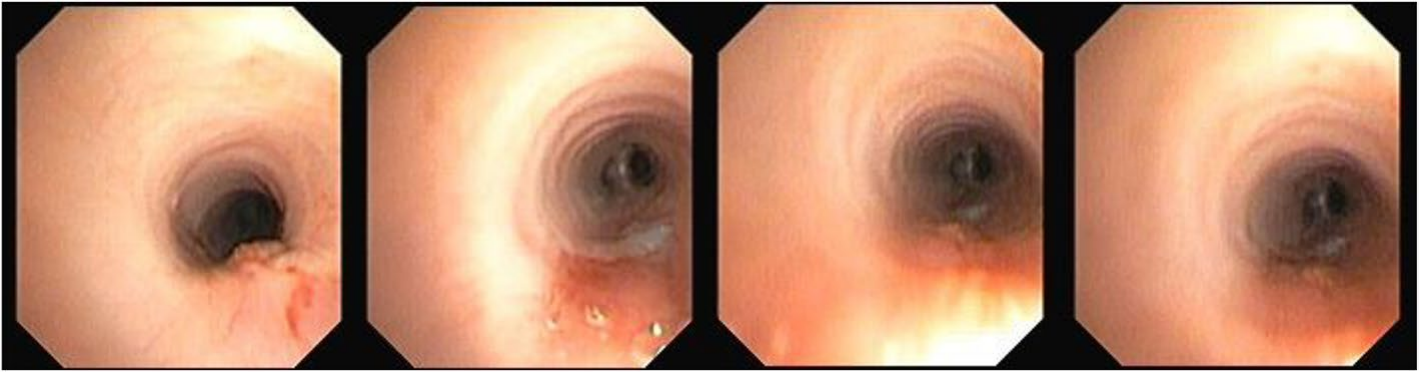
Bronchoscopic view after the surgical repair of the double H-type tracheoesophageal fistula (case 1)

The first case was a 54-day-old infant boy who was referred to Mofid Children’s Hospital, a tertiary center in Tehran province, for the repair of TEF. He was the second child of consanguineous parents (G2P2L2Ab0) with normal prenatal and perinatal history. He was born term through normal vaginal delivery with the birth weight of 3500 g. During the first few days of life, he had cough and spells of cyanosis, resulting in her admission in the Neonatal Intensive Care Unit (NICU). Two weeks later, he re-admitted in the hospital with pneumonia, and received antibiotic therapy. Since his feeding difficulty continued and his pneumonia was not completely resolved with proper antibiotic therapy, he was referred to Mofid Children’s Hospital for further evaluations. After consultation with a pediatric pulmonologist, a double H-type fistula, laryngotracheoesophageal cleft (LTEC) grade 2, and tracheomalacia were detected in fiber optic bronchoscopy (Figs. 1 and 2). The patient was transferred to the pediatric surgery ward. However, during the surgical exploration via a single cervical incision two distinct fistula identified and dissected. During the post-operative course, feeding was started through gastrostomy and was well tolerated. Nonetheless, he developed to poor feeding, fever, tachypnea, and mottling compatible with refractory sepsis with Klebsiella, 10–12 days post operation. During the hospital course, through evaluations were performed and the related data have been summarized in Tables 1 and 2. Therapeutic measures were performed by a pediatric intensivist and a pulmonologist, but he expired on the 13th day post operation. The parents did not assent for an autopsy after death.

**Table 1:** The patient’s laboratory findings through the course of hospitalization

Investigations	Results
White Blood Cells (WBC) (*10^6^/L)	7.9
Hemoglobin (g/dL)	8.7
Platelet (*10^6^/L)	171
Neutrophil (%)	80.4
Lymphocyte (%)	15.1
Sodium (mEq/L)	137
Potassium (mEq/L)	4.9
Calcium (mg/dL)	6.4
Creatinine (mg/dL)	0.87
Blood Urea Nitrogen (BUN) (mg/dL)	40.2
Albumin (g/dL)	2.2
Total protein	4.4
Magnesium (mg/dL)	1.7
C-Reactive Protein (CRP) (mg/dl)	19
Tracheal culture	Klebsiella growth

**Table 2:** Arterial blood gas (ABG) findings before the TEF repair and after becoming ill and toxic

ABG parameters	1st day of admission	10th day post operation	11th day post operation	13th day post operation
PH	7.32	7.07	7.07	6.82
PO2	107.2	58.8	32.7	41.8
PCO2	44	86.7	87.4	58.4
HCO3	23	25.3	25.4	9.7

## Case 2

**Fig. 3:**
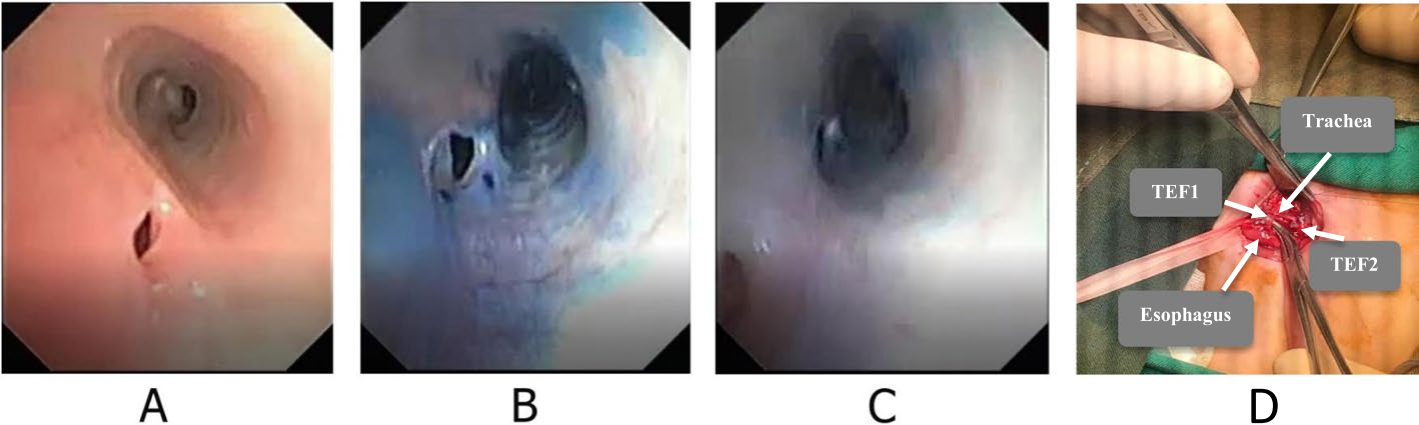
**A** Proximal TEF in bronchoscopy before the surgical repaire in case 2. **B** Distal TEF in bronchoscopy with methylene blue. **C** Proximal and distal TEF in modified bronchoscopy with methylene blue. **D** Both TEF during surgery

A 2780 g newborn girl at the gestational age of 38 weeks with a breech position was born via cesarean section of a diabetic mother (G1P1L1Ab0) who was on metformin during her pregnancy. Her first minute Apgar score was 10 out of 10. Consequently, she was transferred to the rooming ward. At the sixth hour of birth, she developed cough, tachypnea, cyanosis, and choking after breastfeeding. Therefore, she was transferred to the neonatal ward of a local hospital in Qom city, Qom province. On the first day of admission, tachypnea and bilateral pulmonary rales were the only findings in physical examination. On the second day of admission, she developed choking after feeding through gavage. Hence, she was admitted in NICU. She was suspicious to TEF, but the primary evaluations were unremarkable. The patient was hospitalized for 34 days and during the course of hospitalization, she had insufficient weight gain and several episodes of cough and tachypnea following oral feeding. Further evaluations with upper gastrointestinal series only showed stricture in the second part of the duodenum. Based on this finding, pyloromyotomy was done for her. After 72 hours, oral feeding was started for the patient, but cough and choking occurred again. Therefore, flexible bronchoscopy was performed, revealing no findings in favor of TEF. On the 70th day of hospitalization, the patient was transferred to Mofid Children’s Hospital for further evaluations, as none of her complaints was relived. Before operation, the patient was evaluated para clinically (Table 3). In fiber optic bronchoscopy, two separate TEFs and tracheamalacia were found. Finally, both fistulas were repaired via a single cervical incision (Fig. 3). After the operation, she had an acceptable and unremarkable hospital course. She was discharged from the hospital and was regularly followed by a pediatric pulmonologist and a pediatric surgeon. After about 6 months, she is doing well.

**Table 3:** The laboratory findings of the second case

Investigations	Results
WBC (*10^6^/L)	11.9
Hb (g/dL)	12.1
Platelet (*10^6^/L)	313
Neut (%)	55
Na (mEq/L)	133
K (mEq/L)	4.9
Ca (mg/dL)	9.1
Cr (mg/dL)	0.4
Albumin (g/dL)	2.2
CRP	23
CSF culture	No growth
Blood culture	No growth
Blood gas pH	7.52
Blood gas PCO2	40.5
Blood gas HCO3	33.4

## Discussion and conclusion

H-type TEF is one of the congenital esophageal anomalies, which presents with choking during feeding, recurrent coughs, and respiratory infections [2]. Upper gastrointestinal contrast study and tracheobronchoscopy can confirm the diagnosis in case of clinical suspicion [2, 3]. Yet, non-specific symptoms, rare prevalence, and transient obstruction of fistula with small orifices make the early diagnosis of H-type TEF a challenge [3].

Tracheomalacia (TM) is the most prevalent congenital tracheal abnormality with the incidence of 1 per 2100 children. It has been defined as an increased compliance of trachea, leading to dynamic or static collapse [5, 6]. This congenital abnormality presents with non-specific symptoms and can be concomitant with other congenital disorders [7, 8]. Considering the common embryologic origin of trachea and esophagus, TM is a common respiratory problem in esophageal atresia and TEF [9, 10]. A LTEC refers to the congenital malformation of the posterior part of the larynx that establishes an abnormal connection between the laryngotracheal axis and the pharyngoesophageal axis [11]. This abnormality has four types based on the Benjamin and Inglis classification system [12] and its prevalence has been reported as 1 per 10,000–20,000 live births [13]. Although most LC cases are sporadic, some are associated with other congenital abnormalities like TEF [14].

Presence of two isolated H-type TEFs is an extremely rare condition, but has been reported in previous studies. In such cases, some surgeons repaired both fistulas simultaneously in one operation [15], the challenging we face with it in developing countries. Surgery is infact the main treatment [3], with the success rate depending on the skills and pre-operative identification of the fistula level [15]. Hence, most pediatric surgeons use rigid bronchoscopy before selecting the surgical approach [16]. The survival rate after surgical therapy has been reported to range from 70 to 100% [2]. In addition, post-operative complications included vocal cord dysfunction due to recurrent laryngeal nerve palsy and respiratory difficulty requiring tracheostomy [1, 2]. Cuesta et al. investigated the course of diagnosis and treatment of three cases of H-type TEF that had been diagnosed in the first 30 days of their lives. Two of these patients were evaluated via Video Fluoroscopic Swallowing Study (VFSS) and the diagnosis was confirmed by rigid tracheobronchoscopy. After the surgical operations, the patients were hospitalized in intensive care units for 7 days. Surgical complication was reported in one patient who developed pneumothorax because of partial suture rupture. All patients were followed up for 3 months to 2 years, revealing no symptom recurrence and normal feeding and growth [3].

Mattei conducted a study on a newborn with double H-type TEF presented with coughing in every feeding. Operation was considered after contrast esophagogram revealed a single TEF. Rigid bronchoscopy through operation also identified another fistula in the more proximal part of the trachea. Surgical repair was done through thoracotomy and each fistula was repaired using a 4F Fogarty balloon catheter. On the seventh day of operation, an esophagogram confirmed no evidence of leakage from each repair and the patient recovered dramatically [16].

In the present cases with double H-type TEFs, both fistulas were repaired simultaneously in a surgery thorough a cervical incision. The success of the operations was confirmed by tracheobronchoscopy, but one of the patients deteriorated 12 days after the surgery, developing to sepsis and expired unfortunately. However, increasing awareness about the possibility of the presence of a second fistula in the context of H-type TEF is very important among physicians. The issue that, along with the presence of skilled surgeon, is one of the challenges of many developing countries.

## Conclusion

Double H-type tracheoesophageal fistula is a rare type of congenital esophageal anomalies that presents with non-specific signs and symptoms in infants and may be associated with other congenital tracheaesophageal anomalies. This anomaly can be repaired through surgical intervention with high surveillance, but accompanying abnormalities can affect the prognosis.

## Data Availability

The datasets used and/or analyzed during the current study are available from the corresponding author on reasonable request.

## Abbreviations

TEF: Tracheoesophageal Fistula
TM: Tracheomalacia
LTEC: Laryngotracheoesophageal Cleft
NICU: Neonatal Intensive Care Unit
WBC: White Blood Cells
BUN: Blood Urea Nitrogen
CRP: C - reactive protein
AST: Aspartate Aminotransferase
ALT: Alanine Aminotransferase
VFSS: Video Fluoroscopic Swallowing Study
